# Musicokinetic and exercise therapies decrease the depression level of elderly patients undergoing post-stroke rehabilitation: The moderating effect of health regulatory focus

**DOI:** 10.3389/fpsyg.2022.889510

**Published:** 2022-08-15

**Authors:** Li Zhao, Xiaokang Lyu, He Jiang, Xinhai Gao

**Affiliations:** ^1^Zhou Enlai School of Government, Nankai University, Tianjin, China; ^2^Shibei Hospital, Shanghai, China

**Keywords:** musicokinetic therapy, exercise therapy, post-stroke depression, health regulatory focus, rehabilitation

## Abstract

This study aims to investigate the impact of musicokinetic and exercise therapies on the depression level of elderly patients undergoing post-stroke rehabilitation and its possible moderators, the promotion focus (i.e., achieve gains) and prevention focus (i.e., avoid losses or non-gains), which are the two motivational orientations of health regulatory focus. An eight-week randomized controlled trial was employed. Sixty-five elderly patients undergoing post-stroke rehabilitation in a hospital in Shanghai, China. Patients were randomly assigned to the musicokinetic (*n* = 32) therapy group or the exercise (*n* = 33) therapy group. The Mini-mental State Examination Scale measuring the patients’ cognitive functions was used to screen participants. The Hamilton Depression Rating Scale and the Health Regulatory Focus Scale were applied to assess their levels of depression and health regulatory focus on weeks 0, 4, and 8, respectively. The musicokinetic therapy had a significantly better effect than the exercise therapy for individuals who had a lower level of prevention focus, whereas the exercise therapy had a significantly better effect than the musicokinetic therapy for individuals who had a higher level of prevention focus. Musicokinetic therapy and exercise therapy were both effective in decreasing post-stroke depression for elderly patients. But it is important to choose an appropriate type of therapy per the health regulatory focus of elderly patients with post-stroke rehabilitation.

## Introduction

The population is aging rapidly all over the world (Bloom et al., 2015; He et al., 2016). The health, happiness, and wellbeing of elderly patients should be attended to. Stroke is one of the major dangers that threaten the health of aging people. Among stroke patients, the incidence rate of post-stroke depression (PSD) is as high as 32.9–35.9% (Kim et al., 2011; Shi et al., 2017). The PSD exacerbates the suffering and burden of patients and their families, hinders the process of stroke treatment and rehabilitation, affects the prognosis of stroke, and increases the risk of death. To alleviate the suffering of elderly post-stroke patients, it is important to mitigate their PSD simultaneously when they are seeking rehabilitation treatments.

Researchers have investigated the possible mechanisms of depression induced by a stroke from the perspective of psychology to help post-stroke patients and their families. They have found that the self-perceived sense of uselessness, which is an important component of individuals’ understanding of their own aging process, due to dependence on others is the most severe and persistent risk factor for PSD (Singh et al., 2000). Post-stroke patients are more dependent on others because of their reduced mobility and ability to take care of themselves. Some of these patients have difficulty accepting their disabilities. Thus, strong psychological stress reactions, which can induce depression, suicide, or many other serious mental illnesses, are easily triggered (Chiu et al., 2013). Therefore, under the physical and psychological influences on post-stroke patients, the incidence of post-stroke depression (PSD), the common complication of stroke, remains high (Shi et al., 2017). Unfortunately, the sense of uselessness is one the most common negative afflictions experienced by the elderly population (Gu et al., 2016). Thus, the depression level of elder adults recovering from stroke is usually more severe than those of others (Ellis et al., 2010; Zhao et al., 2017). Therefore, the PSD has to be relieved for the elderly patients undergoing post-stroke rehabilitation.

Previous studies have found that music (Kim et al., 2011; Hadidi et al., 2017; Li et al., 2020; Sumakul et al., 2020) and exercise (Knapen et al., 2015; VanDerwerker et al., 2018) therapies have favorable effects in mitigating PSD. The music therapy takes music as the medium to improve individuals’ physical and psychological states. Doing exercise under the exercise therapy leads to positive changes which can be experienced by individuals, such as increased mobility, increased sense of strength and independence. All these positive changes are helpful to reduce anxiety and depression. Therefore, the combination of music therapy and exercise therapy conjoins the advantages of the two. In recent years, among all possible therapies, musicokinetic therapy (aka, music-exercise therapy), which is a therapeutic technique that combines music and exercise therapies, stands out as a promising one. The musicokinetic therapy is developed by Noda et al. (2003), in which the trampoline with live music performance is employed to improve the clinical condition of patients in a persistent vegetative state. It has been found that under the musicokinetic therapy treatment, patients’ psychological and physiological activities can be changed by listening to music while exercising (Noda et al., 2003; Li et al., 2021). It has both cognitive and behavioral effects on the treatment of brain damage (Noda et al., 2004; Van de Winckel et al., 2004; Magee, 2005). However, the direct evidence to whether musicokinetic therapy can improve the emotional status of elderly post-stroke patients remains lacking (Yi et al., 2013). Additionally, few studies have compared the effectiveness of musicokinetic therapy with that of exercise therapy alone to examine whether the performance of musicokinetic therapy performs better in reducing the PSD. Therefore, the impact of musicokinetic therapy and exercise therapy on the depression level of elderly patients undergoing post-stroke rehabilitation is explored in the present study.

Besides, individuals’ willingness and motivation to recover from a stroke can affect their physical and mental recovery status (Maclean et al., 2000; Espel et al., 2016). Research has found that patients with strong individual health motivation will pay attention to the process of rehabilitation treatment (Whyte et al., 2019). As the treatment process progresses and the body recovers, the level of depression can be decreased, since post-stroke patients rely less on others and thus cultivate self-confidence in the rehabilitation process. By contrast, patients who are deeply depressed tend to focus on the negative aspects of stroke and are less motivated to rehabilitate. It shows that the depression symptoms of patients are closely related to the perception and management of their health condition. Therefore, the health regulatory focus (HRF), which is defined as an individual’s tendency to achieve gains or avoid losses and non-gains in the pursuit of health goals (Higgins, 1997; Gomez et al., 2013; Ferrer et al., 2017; Schmalbach et al., 2017), should be considered as an important personality trait that influences the treatment effects. Specifically, there are two types of health regulatory focus. One is promotion focus, which depicts the tendency to achieve positive health outcomes. The other is prevention focus, which represents the tendency to avoid negative health consequences (Ferrer et al., 2017). For now, there is rare relevant evidence indicating that therapies, including music therapy, exercise therapy, or the combination of both – musicokinetic therapy, are directly linked to individuals’ health regulatory focus. Previous studies have shown that the promotion focus and prevention focus can moderate the relationship between the performance incentives and goal attainment (Shah et al., 1998) and there are relationships between the regulatory focus and depression (Gailliot et al., 2006; Klenk et al., 2011). Although the musicokinetic therapy and exercise therapy are presumably to promote patients’ health status, individuals with high promoting tendency adjust their health strategies by approaching a match with the desired state, and individuals with a high preventing tendency adjust their health strategies by avoiding health-related harm, which means patients have different perspectives to understand and address issues. Therefore, with different motivational drives, the promotion focus and prevention focus may have crucial but different roles in moderating the relationship between the treatment therapies and the decreasing of the level of depression.

In sum, this study aims to test and compare the treatment effects of musicokinetic therapy and exercise therapy on mitigating PSD of elderly patients who are undergoing the process of post-stroke rehabilitation. In the meantime, the moderating roles played by the prevention focus and promotion focus in the relationship between different treatment methods and the depression level are tested. Therefore, four hypotheses are drawn specifically: (1) By adopting the musicokinetic therapy, the depression level of elderly patients undergoing post-stroke rehabilitation decreases; (2) By adopting the exercise therapy, the depression level of elderly patients undergoing post-stroke rehabilitation decreases; (3) The musicokinetic therapy works better than the exercise therapy in alleviating the level of PSD; (4) The prevention focus and promotion focus play moderating roles in the relationship between the treatment and the depression level.

## Materials and methods

### Study design

**Figure 1:**
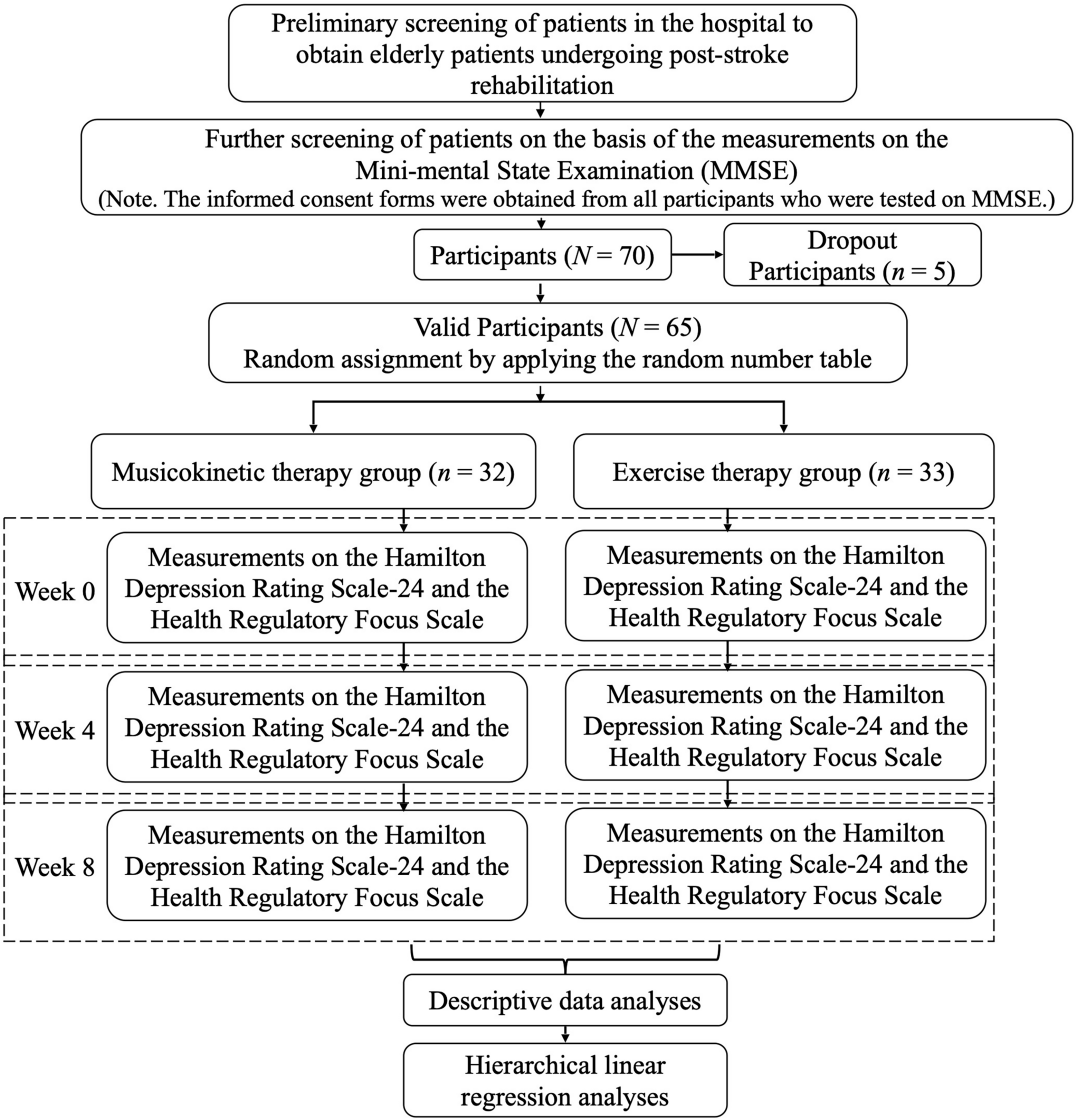
Flowchart of the investigation of the treatment effects of different types of therapy and the moderating role of the health regulatory focus.

This research was designed as a 2 (musicokinetic therapy/exercise therapy) × 3 (Week 0, Week 4, and Week 8) mixed factorial design with treatment method as the between-participants variable and the time point of measurements as the within-participants variable. The flowchart of this study is displayed in Figure 1. First, we screened patients by using inclusion and exclusion criteria (details are in the “Participants” section). Then, we measured the depression level and the level of prevention focus and promotion focus. After data collection, we conducted the descriptive data analyses and the hierarchical linear regression analyses with repeated measurements to figure out how different types of therapy affected the treatment effect of depression and whether the prevention focus and promotion focus play moderating roles in the relationship between the treatment method and the depression level.

### Participants

Considering the particularity of the participant group, we would like to include as many elderly patients undergoing post-stroke rehabilitation treatments as we can reach who are eligible for this study. A convenience sampling method was used, and data were collected from elderly patients undergoing post-stroke rehabilitation treatments in one hospital located in Shanghai, China from December 2019 to March 2020. These patients had symptoms of depression, but their cognitive functions were normal and their degrees of stroke severity were similar. The specific criteria for the participants were as follows:

Inclusion criteria: Patients who (1) were at least 60 years old; (2) met the diagnostic criteria in the 2018 edition of the Chinese Guidelines for the Management of Acute Ischemic Stroke or the 2019 edition of the Chinese Guidelines for the Management of Cerebral Hemorrhage; (3) had approximately two weeks after the stroke; (4) with neurological sequelae requiring rehabilitative exercise therapy; (5) received at least eight points on the Chinese version of Hamilton Depression Rating Scale with 24 items (HDRS-24); and (6) received at least 27 points on the Mini-mental State Examination (MMSE). Exclusion criteria: Patients (1) with other serious organ diseases, such as a malignant tumor, myocardial infarction, and comatose; (2) who were unable to take exercise therapy after the stroke attack; (3) or their relatives who refuse to take the exercise or musicokinetic therapy; (4) who received antidepressant medication in the past month; and (5) who withdrew from treatments due to transfer and personal reasons.

Finally, the valid data collected from 65 participants were included in the final analyses. Five participants were dropped out. Three participants were transferred to another hospital, one participant withdrew, and some data in the experimental records of one participant were missing. The valid rate was 92.86%. Participants were randomly assigned to the musicokinetic (*n* = 32) and the exercise (*n* = 33) therapy groups by using the random number table by the experimenter. The demographic information is shown in Table 1.

**Table 1:** Descriptive statistics for variables (*N* = 65).

Variable	*M*	*SD*
1. Age (years old)	81.14	8.33
	***N***	**%**
2. Gender		
Male	28	43.1
Female	37	56.9
3. Treatment Type		
Exercise Therapy	33	50.8
Musicokinetic Therapy	32	49.2

The study was conducted in accordance with the Declaration of Helsinki (as revised in 2013). In addition, this study was approved by the institutional board of Shanghai Shibei Hospital and Nankai University, and informed consent forms were obtained from all participants and the patients who were tested on the MMSE in the further screening step as shown in Figure 1.

### Measures

The HDRS-24, created by Hamilton (1960), is a widely used depression diagnostic scale (Hamilton, 1960; Trajković et al., 2011). In the present study, the Chinese version of the HDRS-24, a mature questionnaire published in the *Rating Scales for Mental Health* (Wang et al., 1999), was used to measure the level of depression (a list of explanations of the measurement scales used in this article is shown in Appendix A). Participants were measured jointly by two systematically trained clinicians through conversations and observations. The final score was the average of the independent scores provided by clinicians. In the Chinese version of HDRS-24, 14 items were scored on a 5-point Likert scale from 0 to 4, and 10 items were scored on a 3-point scale from 0 to 2, representing from absent to severe, respectively. If the final score was less than 8, the patient could be considered to be normal/non-depressed, and if the final score was 8 or higher, the patient could be considered to have varying degrees of depressive symptoms. In previous studies, the Cronbach’s α ranged from 0.71 to 0.99 (Tang and Zhang, 1984; Liu et al., 2018). In this study, the Cronbach’s α was.92.

In the present study, the MMSE, a widely used test of cognitive function among the elderly, was used to screen and exclude the participants with cognitive impairments. The MMSE was created by Folstein et al. (1975). The Chinese version of the MMSE used in the present study is created by Zhang (1998) and widely used in previous research (Guo et al., 2001; Chen et al., 2019). Participants were instructed to complete tasks, and the obtained scores were based on the task performance. If one task was completed correctly, the participant would receive a score of 1. Otherwise, he/she would receive a score of 0. If the final score was less than 27, which is a widely used standard in rehabilitation studies for stroke patients in China, the patient could be considered to have cognitive impairments.

The Health Regulatory Focus Scale (HRFS) was used to measure the individuals’ prevention and promotion focus in a health-specific context. Twelve items, including six items in each subscale, were contained in the HRFS. All items were scored on a 7-point Likert scale (1 = *strongly disagree*; 7 = *strongly agree*). The higher the score, the more likely the participant was to adopt the strategy. The HRFS was created by Ferrer et al. (Ferrer et al., 2017). We developed the Chinese version of the HRFS. The forward–backward translation was conducted followed by a critical appraisal by an expert panel. A psychologist and a medical scientist were recruited to create the translated Chinese version of HRFS on the basis of the original scale. Two professors in the Department of English at Nankai University, who did not read the original scale, translated the Chinese version of HRFS back into English, which had no significant difference from the original version. Finally, an expert panel consisting of six other psychologists and medical scientists assessed and modified the scale to avoid possible issues, such as ambiguity, difficulty of understanding, and lack of conciseness. The translated version was tested in a Chinese sample of 360 before it was applied in the formal study sample, in which the Cronbach’s α value was 0.85 and 0.81 for the prevention focus and promotion focus, respectively. In previous studies, the Cronbach’s α ranged from 0.70 to 0.85 for the prevention focus and from 0.83 to 0.89 for the promotion focus (Ferrer et al., 2017; Laroche et al., 2019; Gödöllei and Beck, 2020). In the present study, at weeks 0, 4, and 8, the Cronbach’s α values were 0.82, 0.79, and 0.83, respectively, for the prevention focus and 0.85, 0.84, and 0.87, respectively, for the promotion focus.

### Procedure

Out of the 65 valid participants, 32 participants underwent musicokinetic therapy, and 33 participants underwent exercise therapy for two months. All participants ingested one tablet of Sertraline Hydrochloride (50 mg), which is a type of antidepressant drug, every morning during the experimental period.

In the exercise therapy group, the participants focused on active exercises, including isometric muscle, joint function, speech and swallowing function, balance function, and gait training. The length of each exercise was 30 min and performed twice a day. The rehabilitation room in this study was about 80 m^2^. The whole exercise therapy lasted 8 weeks.

In the musicokinetic therapy group, all participants underwent the exercise treatment, which was the same as the participants took in the exercise therapy group, with background music in the rehabilitation room. That is to say, the procedures of the musicokinetic therapy group and the exercise therapy group were identical except for the music component in the musicokinetic therapy group. The Edifier S2000MKIII speaker in the rehabilitation room was used as the music-playing equipment. Participants listened to the same type of ambient pure music from Bandari. Bandari produces music with slower and softer rhythm and many people in China choose to listen to Bandar music to relax. The volume of the music was set to 50 decibels. The musicokinetic therapy lasted for 30 min each time and was performed twice a day. The whole musicokinetic therapy lasted 8 weeks.

The repeated-measures procedure was used to determine the degree of depression (i.e., HDRS-24 score) and the HRF (i.e., HRFS scores) at weeks 0, 4, and 8 after therapy.

### Data analysis

In this study, the descriptive statistical analyses of key variables were performed. The hierarchical linear regression analyses^1^ with repeated measurements were conducted to examine the variables predictive of the participants’ depression level to investigate how different types of therapy affected the treatment effect of depression and whether the HRF played a moderating role in the relationship between the treatment and the depression level. The moderating effects played by the promotion focus and the prevention focus were tested in two different models, since they were two directions of motivation. Furthermore, by testing whether the interaction terms containing treatment type and promotion/prevention focus playing a significant effect in models, the moderating effects could be tested.

## Results

Results of descriptive statistical analyses are presented in Tables 2, 3. It showed that there was a trend of decreasing of the depression level under both groups of musicokinetic therapy and exercise therapy from week 0 to week 8. However, it is worth noting that the depression levels under the musicokinetic treatment were lower than that under the exercise treatment at the first timepoint (will be discussed later). The depression level was negatively correlated with prevention focus, promotion focus, and the treatment type.

**Table 2:** Descriptive statistics for variables (*N* = 65).

Time Point	Group	Variable	25th	Median	75th	Range	*M*	*SD*
Week 0	MT (*n* = 32)	Depression	15.25	20.00	33.75	45.00	23.97	11.33
		Prevention Focus	15.25	22.00	26.50	29.00	20.19	8.18
		Promotion Focus	14.25	22.00	28.00	32.00	21.44	9.46
	ET (*n* = 33)	Depression	17.50	25.00	49.00	54.00	32.30	17.90
		Prevention Focus	6.00	19.00	22.00	30.00	15.76	9.59
		Promotion Focus	6.00	21.00	26.00	32.00	17.61	11.11
	Total	Depression	16.50	21.00	36.00	54.00	28.20	15.49
		Prevention Focus	6.00	20.00	24.00	30.00	17.94	9.13
		Promotion Focus	6.00	22.00	27.50	32.00	19.49	10.43
Week 4	MT (*n* = 32)	Depression	14.00	17.00	24.00	43.00	19.81	10.47
		Prevention Focus	14.00	22.00	25.75	25.00	20.06	7.92
		Promotion Focus	15.25	24.00	28.75	30.00	22.22	9.03
	ET (*n* = 33)	Depression	15.00	24.00	45.50	57.00	30.33	18.47
		Prevention Focus	6.00	20.00	24.00	29.00	16.39	10.15
		Promotion Focus	6.00	20.00	26.00	31.00	16.94	10.32
	Total	Depression	14.50	18.00	31.50	57.00	25.15	15.87
		Prevention Focus	6.00	21.00	25.00	29.00	18.20	9.24
		Promotion Focus	6.50	23.00	27.00	31.00	19.54	9.99
Week 8	MT (*n* = 32)	Depression	10.00	15.00	19.75	40.00	16.38	9.92
		Prevention Focus	16.25	22.00	26.50	24.00	20.03	7.70
		Promotion Focus	19.25	24.00	26.75	28.00	21.94	8.18
	ET (*n* = 33)	Depression	15.00	20.00	44.50	56.00	29.70	19.43
		Prevention Focus	6.00	18.00	22.50	27.00	15.30	9.01
		Promotion Focus	6.00	20.00	26.00	30.00	17.39	10.48
	Total	Depression	11.00	17.00	30.00	59.00	23.14	16.78
		Prevention Focus	6.50	20.00	25.00	27.00	17.63	8.66
		Promotion Focus	7.50	23.00	26.00	30.00	19.63	9.62

MT is the musicokinetic therapy group, and ET is the exercise therapy group.

**Table 3:** Zero-order correlations for the variables (*N* = 65).

	1	2	3	4	5	6
1. Age	–					
2. Depression	0.075	–				
3. Prevention Focus	−0.025	−0.681^***^	–			
4. Promotion Focus	0.019	−0.660^***^	0.740^***^	–		
5. Gender^a^	0.194^**^	0.165^*^	−0.241^**^	−0.224^**^	–	
6. Treatment Type^b^	−0.302^***^	−0.334^***^	0.239^**^	0.229^**^	−0.076	–

a Gender was coded as 1 = Male, 2 = Female.

b Treatment type was coded as 1 = Exercise therapy, 2 = Musicokinetic therapy.

* *p* < 0.05;

** *p* < 0.01;

*** *p* < 0.001.

Hierarchical linear regression analyses with repeated measurements were conducted to examine the variables predictive of the participants’ depression level using SPSS 24.0. Before the hierarchical multiple regression analysis was performed, the independent variables were examined for collinearity. Results of the variance inflation factor (all less than 2.0) and collinearity tolerance (all greater than 0.75) suggested that the estimated *β* values were well established in the following regression model (a detailed list of explanations of the mathematical symbols used in this article is shown in Appendix B). It was shown that the depression level of elderly patients undergoing post-stroke rehabilitation significantly decreases under both the musicokinetic treatment group (*M*_week0_ = 23.97, *M*_week4_ = 19.81, *M*_week8_ = 16.38) and the exercise treatment group (*M*_week0_ = 32.30, *M*_week4_ = 30.33, *M*_week8_ = 29.70), *F*(2, 62) = 64.62, *p* < 0.001, *F*(2, 64) = 5.627, *p* = 0.006, respectively. Therefore, the first and second hypotheses were validated. Comparing the levels of depression at all three time points under the musicokinetic treatment and the exercise treatment, it was found that the depression levels under the musicokinetic treatment were significantly lower than that under the exercise treatment, *F*(2, 126) = 11.26, *p* < 0.001. The third hypothesis was validated. However, as mentioned above, the depression level that was measured at the first time point of the musicokinetic treatment group was significantly lower than that of the exercise treatment group, *t*(63) = 2.24, *p* = 0.029, 95% CI = [0.88, 15.78]. To further compare the effects between musicokinetic and exercise treatments without the consideration of the influence of health regulatory focus, a hierarchical multiple regression analysis was conducted, with depression level as the dependent variable, gender, age, and measurement timepoint were entered in the first block, treatment type was entered in the second block in the model. It was found that after controlling for gender, age, and measurement timepoint, musicokinetic therapy performed better than exercise therapy in reducing depression level, Δ*F* = 5.357, *B* = −0.339, *SE_B_* = 2.254, *p* < 0.001, *pr* = −0.334. But still, future studies should be cautious when using this conclusion.

**Figure 2:**
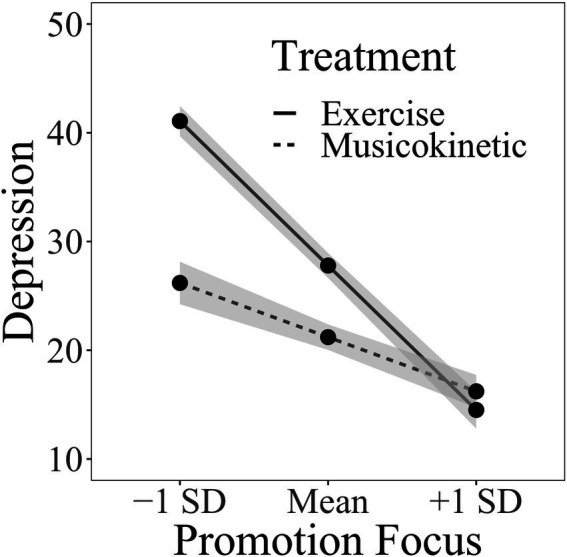
Interaction between the promotion focus and the treatment type.

For the test of the moderating effect of the promotion focus between treatment type and depression level, age and gender were entered into the first block, which means that in the analyses of the effect of treatment type, health regulatory focus, and timepoints of repeated measures, the influence of age and gender was controlled. The treatment type, promotion focus, and time with dummy coding were entered into the second block. The 2-way interactions between the variables in the second block were entered into the third block. The 3-way interactions among the variables in the second block were entered into the fourth block. The interaction between different time points was constant and not included. Regression coefficients with *p* value smaller than 0.05 were considered statistically significant to show possible predictive effects. The combination of gender and age did not account for a significant amount of variation in individuals’ depression levels (Table 4). No significant 3-way interaction was observed in the treatment type, promotion focus, and different measurement time points. However, a significant interaction between the treatment type and promotion focus was observed. Figure 2 depicts the simple slopes relating promotion focus to the depression level under the condition of two different treatment types. At any given treatment type, the increased promotion focus was associated with decreased depression levels. However, the exercise therapy was getting a significantly stronger effect than the musicokinetic therapy with promotion focus increasing. In addition, as shown in Table 4, in the presence of other variables, compared with that at week 0, a significant decrease in the depression level at week 8 was observed. However, no significant difference in the depression level was observed between weeks 0 and 4 and between weeks 4 and 8 (Δ*F* = 0.550, *B* = 0.143, *SE_B_* = 6.580, *p* = 0.459, *pr* = 0.055).

**Table 4:** Summary of hierarchical regression analysis for predicting the depression level – promotion focus (*N* = 65).

Variable	*B*	*SE_B_*	Δ*F*	*pr*	*df_1_*	*df_2_*	*R^2^*	Adj *R^2^*	Δ*R^2^*
Step 1			2.881		2	192	0.029	0.019	0.029
Age	0.045	0.140	0.386	0.045					
Gender	0.156	2.352	4.640^*^	0.154					
Step 2			42.146^***^		4	188	0.488	0.472	0.459
Treatment Type	−0.183	1.816	10.467^**^	−0.230					
td2	−0.088	2.053	2.135	−0.106					
td3	−0.144	2.054	5.748^*^	−0.172					
Promotion Focus	−0.617	0.090	123.989^***^	−0.630					
Step 3			5.949^***^		5	183	0.560	0.533	0.072
Treatment Type × Pro	1.074	0.169	27.277^***^	0.360					
Treatment Type × td2	−0.035	3.968	0.034	−0.014					
Treatment Type × td3	−0.179	3.953	0.914	−0.047					
Pro × td2	−0.038	0.196	0.082	−0.021					
Pro × td3	−0.109	0.199	0.669	−0.060					
Step 4			0.218		2	181	0.561	0.529	0.001
Treatment type × Pro × td2	0.239	0.399	0.274	0.039					
Treatment type × Pro × td3	0.275	0.411	0.356	0.044					

Promotion focus is noted as “Pro.” “td2” means the comparison between weeks 0 and 4. “td3” means the comparison between weeks 0 and 8. “*pr*” is the partial correlation.

* *p* < 0.05;

** *p* < 0.01;

*** *p* < 0.001.

**Figure 3:**
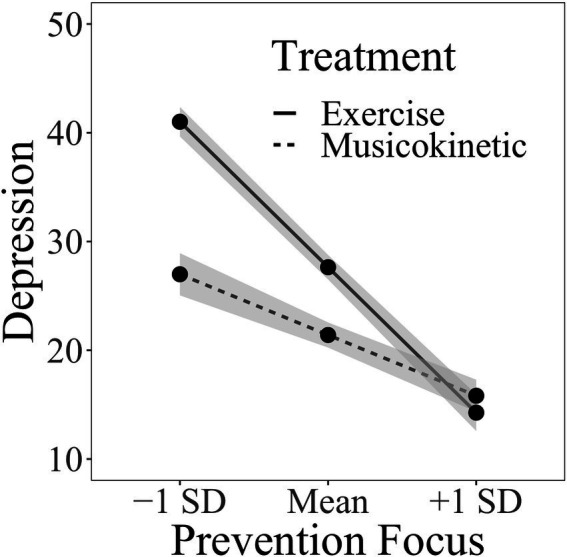
Interaction between the prevention focus and the treatment type.

For the test of the moderating effect of the prevention focus between the treatment type and the depression level, age and gender were entered into the first block. The treatment type, prevention focus, and time were entered into the second block. Their 2-way interactions were entered into the third block, and their 3-way interactions were entered into the fourth block. The combination of gender and age did not account for a significant amount of variation in the individuals’ depression levels (Table 5). No significant 3-way interaction was observed in the treatment type, prevention focus, and different measurement time points. However, a significant interaction was observed between the treatment type and the prevention focus. Figure 3 depicts the simple slopes relating prevention focus to depression level at two treatment types. As shown in Figure 3, at any given treatment type, the increased prevention focus was associated with decreased depression levels. However, the exercise therapy was getting a significantly stronger effect than the musicokinetic therapy with promotion focus increasing. In addition, as shown in Table 5, in the presence of other variables, compared with that at week 0, the depression level at week 8 significantly decreased. However, no significant difference in the depression level was observed between weeks 0 and 4 and between weeks 4 and 8 (Δ*F* = 1.781, *B* = −0.078, *SE_B_* = 2.002, *p* = 0.184, *pr* = −0.097).

**Table 5:** Summary of hierarchical regression analysis for predicting depression level – prevention focus (*N* = 65).

Variable	*B*	*SE_B_*	Δ*F*	*pr*	*df_1_*	*df_2_*	*R^2^*	*Adj R^2^*	Δ*R^2^*
Step 1			2.881		2	192	0.029	0.019	0.029
Age	0.045	0.140	0.386	0.045					
Gender	0.156	2.352	4.640	0.154					
Step 2			46.893^***^		4	188	0.514	0.499	0.485
Treatment type	−0.179	1.768	10.589^**^	−0.231					
td2	−0.081	2.001	1.882	−0.100					
td3	−0.159	2.001	7.325**	−0.194					
Prevention focus	−0.641	0.097	140.600***	−0.654					
Step 3			4.981***		5	183	0.572	0.547	0.058
Treatment type × Pre	0.975	0.184	23.123***	0.335					
Treatment type × td2	−0.166	3.905	0.808	−0.066					
Treatment type × td3	−0.192	3.942	1.062	−0.076					
Pre × td2	0.049	0.214	0.131	0.027					
Pre × td3	−0.043	0.223	0.101	−0.024					
Step 4			0.363		2	181	0.574	0.543	0.002
Treatment type × Pre × td2	0.050	0.441	0.012	0.008					
Treatment type × Pre × td3	0.369	0.456	0.626	0.059					

Prevention focus is noted as “Pre.” “td2” means the comparison between weeks 0 and 4. “td3” means the comparison between weeks 0 and 8. “*pr*” is the partial correlation.

^*^p < 0.05;

** *p* < 0.01;

*** *p* < 0.001.

In summary, the fourth hypothesis has been validated, which means that the prevention focus and the promotion focus play moderating roles in the relationship between the treatment types and the depression level.

## Discussion

Although not all elderly patients who are undergoing post-stroke rehabilitation are experiencing PSD, they are vulnerable to be depressed and difficult to recover from it. In the past, the depression rehabilitation in elderly patients with stroke has not been investigated thoroughly. Our study advances the research in this area one small step forward. According to the results in this study, after controlling for the influence of age and gender, the musicokinetic therapy is effective in reducing the PSD level of elderly patients undergoing post-stroke rehabilitation, especially when comparing the levels of depression between weeks 0 and 8, and this conclusion has not been proven yet by previous experiments. Together with exercise, certain music, as a special way to transmit information, can create a suitable rehabilitation environment for individuals. Patients with PSD who are treated with the musicokinetic therapy may experience mental (i.e., relieved tension, calmness, and improved self-confidence) and physical (i.e., decreased heart rate and blood pressure, improved blood circulation and sleep, and promoted repair of the nervous system) improvements. As a result, the level of depression decreases. Therefore, the musicokinetic therapy, a type of treatment with low investment but high safety, should be promoted in the rehabilitation of patients with stroke.

We also confirmed that the exercise therapy alone can decrease the level of depression for elderly patients with post-stroke rehabilitation, which is consistent with the conclusion in previous studies (Lai et al., 2006; van het Hoofd et al., 2011). Notably, based on the results of the present study, the treatment effect of decreasing the level of depression of the musicokinetic therapy is not always better than that of the exercise therapy. One possible reason may be that elderly individuals are less receptive to external factors, such as music, than young individuals. However, considering that the musicokinetic therapy is also effective, the most appropriate therapy should be chosen on the basis of the patient’s conditions. In practice, the best treatment option should be determined considering the differences among almost all patient situations, such as the physical condition, mental condition, and family class, and many other factors.

As aforementioned, there was a statistically significant difference between the musicokinetic therapy group and exercise therapy group, regarding the baseline of depression level. It means that the depression level of participants included in this study varies in a certain range, and after the randomization grouping process, the level of depression of the musicokinetic treatment group was lower than that of the exercise treatment group. This situation is caused by the random grouping and not by experimenter intervention. After careful examination of the treatment effect of different therapies, it could be concluded that musicokinetic therapy performed better than exercise therapy without considering the moderating effects of promotion focus and prevention focus. To be more specific, in the direct impact from treatment type on depression level, the score of depression decreases quickly under the musicokinetic therapy than that under the exercise therapy. Although the decrease in depression level is relatively small under both treatment conditions, it is clinically meaningful.

Another important finding of this study is that the prevention focus and promotion focus play a moderating role in the effects of different therapies on the depression level separately. When the prevention focus and promotion focus are relatively low, the musicokinetic therapy is more effective than the exercise therapy. When the prevention focus and the promotion focus are relatively high, the exercise therapy is more effective than the musicokinetic therapy. Therefore, patients with different HRFs can be targeted with musicokinetic or exercise therapy to achieve improved treatment effects. However, the threshold of the scores of the prevention focus and promotion focus to determine which type of therapy should be used remains unclear and needs further investigation. Additionally, the health motivation changes with health goals. Choosing the most appropriate type of therapy in the changing health motivation on the basis of the corresponding prevention focus and promotion focus is difficult.

There are several drawbacks we have to mention in this study. First, considering the ethics of the study, the cognitive ability of the participants, and the willingness of cooperation of the patients and their family members, establishing a control group without any post-stroke treatment is difficult. Therefore, whether the depression level can decrease with the duration of stroke naturally remains unclear.

In addition, we have only used the results obtained by evaluating patients by using the MMSE scale as an inclusion criterion to screen participants. The cognitive impairment caused by stroke was not further investigated in the present study. Music and exercise therapies can help improve the cognitive functions of post-stroke patients (Li et al., 2015; Moore et al., 2015; Grau-Sánchez et al., 2020). More direct evidence of cognitive improvement from the use of musicokinetic therapy can further advance the field of stroke rehabilitation and the promotion of musicokinetic therapy.

It is worth noting that this study was conducted at the time of the outbreak of the COVID-19 pandemic in China. Many hospitals used closed-off management during this period. Individuals and the whole society are in a difficult time. In this context, finding suitable patients to be participants for this study is not easy. Therefore, the number of participants can be one of the reasons that the conclusions in this study are limited. Furthermore, the COVID-19 pandemic may have impacts on participants’ mental state (all participants in this study were free of COVID-19), which in turn affect their recovery.

Similar to the impact of COVID-19 on participants’ mental state, their physical state may be influenced by other diseases. Although we have taken some diseases into consideration when screening participants, such as malignant tumor, myocardial infarction, comatose, and other serious diseases, due to the particularity of the elderly patients undergoing post-stroke rehabilitation, it is true that these patients may suffer from other diseases in addition to depression when they are taking musicokinetic therapy or exercise therapy. Especially, depression has many comorbidities, which are closely related to the depression symptoms, such as anxiety, sleep disturbances, cognitive impairment, and so on. These diseases can influence the treatment effects, and the treatments can also impact the comorbidities. More importantly, they all have potential impacts on the depression level. Therefore, it may be one of the reasons that there are differences between the musicokinetic treatment group and the exercise treatment group regarding the level of depression. In the future, researchers should conduct more comprehensive research, in which they should consider the coexistence of disease and its impact on depression.

Previous studies point out that circadian rhythms have impacts on ADHD (Coogan et al., 2016), the circadian rhythms should also have impacts on the depression level. To be more specific, sleep disturbances are closely correlated with depression (Franzen and Buysse, 2008). Wittmann et al. (2018) found that the circadian timekeeping system was linked to depressive disorders, and they used the chronobiological therapy, which was based on the disturbing processes. Therefore, when measuring and interpreting the level of depression, it should take the time of the day into consideration. In the current study, it was not at a specific timepoint to measure the depression level at the day of weeks 0, 4, and 8. Future studies should notice this limitation and measure the depression level at a specific time of the day.

To further validate our findings, future studies should investigate the effect of musicokinetic therapy on post-stroke patients in all age groups. The type of music may also be a factor that can affect the treatment effect of the musicokinetic therapy. In this study, soothing music from Bandari is used. Without testing and comparing with other genres of music, determining whether other genres of music are more effective for rehabilitation is difficult. Additionally, to improve the external validity of the results obtained from this study, we should apply more rigorous constraints to uncontrollable factors and apply full consideration to different situations of patients.

## Conclusion

In conclusion, in this study, whether the musicokinetic and the exercise therapies could decrease the degree of depression of elderly patients undergoing post-stroke rehabilitation was investigated. The moderating effect of the HRF on the relationship between different types of therapy and the depression level was explored. The depression level, prevention focus, and promotion focus of participants were repeatedly measured at weeks 0, 4, and 8. In both types of therapy, the level of depression decreased significantly from week 0 to week 8. For individuals who had a lower level of prevention/promotion focus, the musicokinetic therapy had a significantly better effect than the exercise therapy, whereas, for individuals who had a higher level of prevention/promotion focus, the exercise therapy had a significantly better effect than the musicokinetic therapy. In summary, for the elderly patients undergoing post-stroke rehabilitation, the musicokinetic and exercise therapies decrease the depression level. When the HRF is low, the musicokinetic therapy works better than the exercise therapy. Therefore, the appropriate type of therapy could be selected in accordance with the HRF of elderly patients undergoing post-stroke rehabilitation.

## Data availability statement

The raw data supporting the conclusions of this article will be made available upon request to the corresponding author, without undue reservation.

## Ethics statement

The studies involving human participants were reviewed and approved by the institutional board of Shanghai Shibei Hospital and Nankai University. The patients/participants provided their written informed consent to participate in this study.

## Author contributions

LZ, XL, and XG: conception, design, data analysis, and interpretation. XG and HJ: administrative support. LZ and XG: provision of study materials or patient. XL, HJ, and XG: collection and assembly of data. LZ, XL, HJ, and XG: manuscript writing. All authors contributed to the article and approved the submitted version.

## Funding

This work was supported partially by the Tianjin Social Science Foundation of China (TJJX21-011) and the Developmental Program of Liberal and Social Sciences of Nankai University (ZB22BZ0109). The funders had no role in the study design, data collection and analyses, the decision to publish, or the preparation of the manuscript. Additionally, the funders had no influence on the interpretation of data and the final conclusions drawn.

## Conflict of interest

The authors declare that the research was conducted in the absence of any commercial or financial relationships that could be construed as a potential conflict of interest.

## Publisher’s note

All claims expressed in this article are solely those of the authors and do not necessarily represent those of their affiliated organizations, or those of the publisher, the editors and the reviewers. Any product that may be evaluated in this article, or claim that may be made by its manufacturer, is not guaranteed or endorsed by the publisher.

## Footnotes

^1^The hierarchical multiple regression analysis allows specifying a fixed order of entry for variables to control the effects of covariates and test the effects of certain predictors independent of the influence of others (Ross and Willson, 2017).

## Appendix A

**Table:**

MMSE	Mini-mental State Examination.
HDRS-24	Hamilton Depression Rating Scale with 24 items.
HRFS	Health Regulatory Focus Scale.

## Appendix B

**Table:**

*N*	Total sample size.
*n*	Sample size in the corresponding group.
*M*	Mean.
*SD*	Standard deviation.
*p*	Level of significance.
*F*	Value in a regression analysis.
Δ*F*	Change in *F* value.
*β*	Standardized regression coefficient.
*B*	Unstandardized regression coefficient.
*SE_B_*	Standard error of unstandardized regression coefficient.
*pr*	Partial correlation.
*df*	Degrees of freedom.
*R^2^*	Coefficients of determination.
Δ*R^2^*	Change in coefficients of determination.
